# Supplementary material for: Direct transport vs secondary transfer to level I trauma centers in a French exclusive trauma system: Impact on mortality and determinants of triage on road-traffic victims
Source: PLoS One. 2019 Nov 21;14(11):e0223809. doi: 10.1371/journal.pone.0223809 (PMC6872206; doi:10.1371/journal.pone.0223809)
Supplement: S1 File — (DOCX) [file pone.0223809.s001.docx]

**Supplemental material 1: Database linking process**

The linking process of the two databases (Traumabase® and RAAR) was performed by programming a 3 steps procedure on R software (R Development Core Team (2008). R Foundation for Statistical Computing, Vienna, Austria. URL http://www.R-project.org):

1/ Identifying variables containing shared information between the two databases (table below):

| **RAAR files** | **Traumabase® registry** | **Variable function** |
| --- | --- | --- |
| Date of accident | Date of admission | Merging |
| Month/Year of birth | Day/Month/Year of birth | Merging |
| Gender | Gender | Merging |
| Type of vehicle and type of user | Mechanism of injury | Merging |
| Department of accident | Department of EMS providers | Merging |
| Socio-professional activity | Socio-professional activity | Complementary |
| Day-30 mortality status | Day-30 mortality status | Complementary |

As 30-day mortality status was the principal outcome of the study, this criterion was not taken into account in our merging algorithm. Complementary variables were available for manual matching.

2/ Then, for each patient of Traumabase registry:

a/ Computing a distance from each potential RAAR patients, i.e. whose accident occurred within 2 days before date of hospital admission.

Distance between a Traumabase® patient and a RAAR patient corresponded to the probability that these patients were not retained for match, according to a logarithmic model involving differences in values of shared variables. Weighting coefficients of each variable differences were defined:

- At the beginning of the process, according to a shared experience with RAAR users: lower weights for known frequent errors (e.g. non matching departments, one day difference between date of accident and date of admission) and higher weights for less plausible errors (e.g. opposite sex, differences in date of birth in terms of string distance),
- Then updated through an automatic learning algorithm consisting of a logistic predictive model based on previous decisions to match or not (manual match). The stability of coefficients values over ongoing process was graphically assessed.

b/ Performing an automatic match when there was only one potential RAAR candidate, defined by a zero distance from Traumabase® index patient *(the distance threshold initially set to zero, could then be adjusted by the data manager, but was never done).*

c/ Otherwise proposing eligible patients, defined by a distance below 0.6, and allowing a *manual match* if the data-manager considered that the patients are identical.

3/ Repeating the entire process twice and comparing the results.

The quality of this match process was globally assessed by two-by-two confrontation of shared variables values.
