# Supplementary material for: Direct transport vs secondary transfer to level I trauma centers in a French exclusive trauma system: Impact on mortality and determinants of triage on road-traffic victims
Source: PLoS One. 2019 Nov 21;14(11):e0223809. doi: 10.1371/journal.pone.0223809 (PMC6872206; doi:10.1371/journal.pone.0223809)
Supplement: S2 File — Complete cases included 3868 patients in the direct group and 262 in the secondary transfer), SAP remained in the final model but not HR and SpO2. As it can be seen by comparing to Table 4, the values of the OR of all the identified predictors were marginally modified in this sensitivity analyses. Data missing not at random are known to introduce bias, so we chose to withdraw these prehospital variables and we showed that it had no meaningful impact on our results with these sensitivity analyses. (DOCX) [file pone.0223809.s002.docx]

**Table 3**. Results of the multivariate analysis to identify variables associated to the secondary transfer

| **Variable** | **OR, CI 95%** | **p** | **Overal**  **test F** |
| --- | --- | --- | --- |
| **Intercept** |  | <0.001 |  |
| **SAP prehospital** | 1.01 [1.00 – 1.01] | 0.01 |  |
| **Age** |  |  | 0.01 |
| [0,17] | 1.9 [1.2-3.] | 0.01 |  |
| ]17,44] | 1 | - |  |
| ]44,64] | 1.6 [1.1 – 2.2] | <0.01 |  |
| ]64, 100] | 1.8 [1.1 - 2.8] | 0.01 |  |
| **Mecanism** |  |  | 0.01 |
| MVA | 1 | - |  |
| Bicycle | 2.5 [1.5 - 4.2] | <0.001 |  |
| Pedestrian | 1.6 [1.0 - 2.4] | 0.04 |  |
| Motorcycle | 1.3 [0.9 - 1.8] | 0.20 |  |
| Other | 2.0 [0.8 - 4.2] | 0.11 |  |
| **Entrapment** | 0.8 [0.5 - 1.3] | 0.45 |  |
| **Speed** | 0.6 [0.4 - 0.7] | <0.001 |  |
| **GCS initial*** | 1.4 [1.3 - 1.5] | <0.001 |  |
| **AC/AP therapy** | 2.0 [1.1 - 3.3] | 0.01 |  |
| **AIS head ≥ 3** | 3.0 [2.0 - 4.3] | <0.001 |  |
| **AIS face*** | 0.8 [0.6 – 1.0] | 0.05 |  |
| **AIS abdomen ≥ 3** | 4.1 [2.9 - 5.9] | <0.001 |  |
| **AIS pelvis ≥ 3** | 0.5 [0.4 - 0.7] | <0.001 |  |
| **AIS head ≥3 * AIS abdomen ≥ 3** | 0.2 [0.04 - 0.5] | <0.001 |  |

**OR, CI 95%:** Odds ratio and its 95% confidence interval

**SAP:** Systolic arterial pressure, **MVA**: motor vehicle accident, **GCS**: Glasgow coma scale, **AC/AP therapy**: anticoagulant or antiplatelet therapy, **AIS**: Abbreviated Injury Scale

**Severe head trauma**: head AIS ≥ 3

**Severe abdominal trauma**:

* Continuous variables

Akaike criteria: 1746

Hosmer Lemeshow: p=0.65

AUC Roc curve: 0.75 [0.72-0.79]
