# Supplementary material for: Direct transport vs secondary transfer to level I trauma centers in a French exclusive trauma system: Impact on mortality and determinants of triage on road-traffic victims
Source: PLoS One. 2019 Nov 21;14(11):e0223809. doi: 10.1371/journal.pone.0223809 (PMC6872206; doi:10.1371/journal.pone.0223809)
Supplement: S1 Table — BMI: body mass index, ASA: American society of anesthesiologists, AC/AP therapy: anticoagulant and/or antiplatelet therapy, MVA: motor vehicle accident, SAP: systolic arterial blood pressure, HR: Heart rate, SpO2: peripheral oxygen saturation, min: minimal, max: maximal. (DOCX) [file pone.0223809.s003.docx]

**Table S1.** Prehospital demographic, physiologic and injury characteristics of patients according to the 30 days mortality outcome.

|  | **Dead day 30**  **(n= 278)** | **Survivors day 30**  **(n= 4134)** | **p** |
| --- | --- | --- | --- |
| **Demography & outcome** | | | |
| **Age (year)** | 48 (23) | 37 (17) | < 0.001 |
| **Male (%)** | 202 (73%) | 3209 (78%) | 0.05 |
| **BMI (kg/m^2^)** | 25 (6) | 25 (5) | 0.89 |
| **ASA**   - **1** - **2** - **≥ 3** | 147 (61%)  69 (29%)  24 (10%) | 2989 (75%)  906 (23%)  84 (2 %) | < 0.001 |
| **AC/AP therapy** | 25 (9%) | 161 (4%) | < 0.001 |
| **Professional situation**   - **Working** - **Student** - **No activity** - **Other** | 68 (39%)  22 (13%)  74 (43%)  8 (5%) | 2061 (64%)  442 (14%)  595 (18%)  128 (4%) | < 0.001 |
| **Accident Characteristics** | | | |
| **Mechanism of injury (all blunt)** |  |  | < 0.001 |
| - **MVA** - **Motorbike** - **Bicycle** - **Pedestrian** - **Other** | **80 (29%)**  **77 (28%)**  **16 (6%)**  **95 (34%)**  **10 (4%)** | **1361 (33%)**  **1823 (44%)**  **196 (5%)**  **656 (16%)**  **98 (2%)** |  |
| **Area of accident**   - **Inner region** - **Outer region** | **140 (50%)**  **138 (50%)** | **2233 (54%)**  **1901 (46%)** | **0.26** |
| **Ejection** | **75 (46%)** | **1340 (42%)** | **0.39** |
| **Global assessment of speed** | **177 (75%)** | **2640 (72%)** | **0.27** |
| **Death in the same vehicle** | **6 (4%)** | **101 (3%)** | **0.93** |
| **Traumatic cardiac arrest** | **99 (36%)** | **27 (0.7%)** | < 0.001 |
| **Prehospital variables** | | | |
| **SAP min (mmHg)** | **107 (45)** | **130 (24)** | < 0.001 |
| **HR max (beats/min)** | **87 (36)** | **88 (19)** | **0.22** |
| **Glasgow Coma Scale** | **3 [3 - 10]** | **15 [14 - 15]** | < 0.001 |
| **SpO2 min (%)** | **94 [80 – 98]** | **98 [96 - 100]** | < 0.001 |

**BMI:** body mass index, **ASA:** American society of anesthesiologists, **AC/AP therapy:** anticoagulant and/or antiplatelet therapy, **MVA:** motor vehicle accident, **SAP:** systolic arterial blood pressure, **HR:** Heart rate, **SpO_2_ :** peripheral oxygen saturation, **min:** minimal, **max:** maximal.
