# Supplementary material for: Direct transport vs secondary transfer to level I trauma centers in a French exclusive trauma system: Impact on mortality and determinants of triage on road-traffic victims
Source: PLoS One. 2019 Nov 21;14(11):e0223809. doi: 10.1371/journal.pone.0223809 (PMC6872206; doi:10.1371/journal.pone.0223809)
Supplement: S2 Table — SOFA: sequential organ failure assessment, SAPS II: simplified acute physiologic score, ISS: injury severity score, AIS: abbreviated injury scale, SAP: systolic arterial blood pressure, ICU: intensive care unit, LOS: length of stay. Hemorrhagic shock: receiving ≥ 4 packed red blood cells concentrate within 6 hours. Severe head trauma: Glasgow coma scale ≤ 8 and head AIS > 1. (DOCX) [file pone.0223809.s004.docx]

**Table S2.** Severity, clinical characteristics and hospital course of trauma patients according to the 30 days mortality outcome.

|  | **Dead**  **(n= 278)** | **Survivors day 30**  **(n=4134)** | **p** |
| --- | --- | --- | --- |
| **Severity of injuries** | | | |
| **SOFA* (day 1)** | 11 [8 – 14] | 0 [0 – 2] | < 0.001 |
| **SAPS II* (day 1)** | 65 [52 – 76] | 15 [9 – 26] | < 0.001 |
| **ISS*** | 35 [26 – 45] | 10 [5 – 19] | < 0.001 |
| **Head and neck AIS ≥ 3** | 209 (76%) | 794 (19%) | < 0.001 |
| **Face AIS ≥ 2** | 69 (25%) | 428 (10%) | < 0.001 |
| **Thorax AIS ≥ 3** | 153 (55%) | 1115 (27%) | < 0.001 |
| **Abdomen AIS ≥ 3** | 58 (21%) | 426 (10%) | < 0.001 |
| **Extremities pelvis AIS ≥ 3** | 94 (34%) | 1079 (26%) | 0.01 |
| **At admission** | | | |
| **Total pre-hospital time* (min)** | 80 [56 – 100] | 70 [53 – 100] | 0.038 |
| **SAP (mmHg)** | 106 (45) | 130 (24) | < 0.001 |
| **Hemoglobin (g/dL)** | 10.9 (2.4) | 13.4 (1.9) | < 0.001 |
| **Lactate (mmol/L)** | 5.7 (4.7) | 2.2 (1.5) | < 0.001 |
| **Prothrombin rate (%)** | 51 (25) | 84 (16) | < 0.001 |
| **Surgery first 24h** | 93 (34%) | 2000 (48%) | < 0.001 |
| **Specific type of injuries** | | | |
| **Hemorrhagic shock** | 96 (35%) | 184 (5%) | < 0.001 |
| **Severe head trauma** | 158 (57%) | 251 (6%) | < 0.001 |
| **Spine trauma** | 50 (21%) | 477 (12%) | < 0.001 |
| **Medullary injury** | 26 (9%) | 112 (3%) | < 0.001 |
| **Evolution and outcome** | | | |
| **Infection** | 27 (41%) | 165 (20%) | < 0.001 |
| **ICU LOS** | 3 [1 - 7] | 2 [2 – 5] | < 0.001 |
| **Hospital LOS** | 3 [1 – 6] | 8 [3 – 18] | <0.001 |

**SOFA:** sequential organ failure assessment, **SAPS II:** simplified acute physiologic score, **ISS:** injury severity score, **AIS:** abbreviated injury scale, **SAP:** systolic arterial blood pressure, **ICU:** intensive care unit, **LOS:** length of stay.

**Hemorrhagic shock**: receiving ≥ 4 packed red blood cells concentrate within 6 hours.

**Severe head trauma**: Glasgow coma scale ≤ 8 and head AIS > 1.
