# Supplementary material for: Direct transport vs secondary transfer to level I trauma centers in a French exclusive trauma system: Impact on mortality and determinants of triage on road-traffic victims
Source: PLoS One. 2019 Nov 21;14(11):e0223809. doi: 10.1371/journal.pone.0223809 (PMC6872206; doi:10.1371/journal.pone.0223809)
Supplement: Attachment — Submitted filename: PLOS review.docx [file pone.0223809.s005.docx]

Trauma data base retrospective analysis of direct versus secondary road trauma admissions.

Primary outcome: mortality 30 days

English: good

Aim: interesting

Limits acknowledged.

My main concern is the small number of patients who died (primary outcome) in secondary admissions group (9!). A statistical consultant opinion is warranted to ensure these results are sound and assessable .

Suggestions:

Abstract:

Define groups in methods and do not explain again in results

Mention confounders

Define undertriage in methods and report in results

Body

MGAP has been introduced twice

In results:

Add number and % of deaths in both groups. It is relevant as the number is secondary transfer group are really small

Again stick to primary and secondary admission group. It is too often re introduced

There is a typo in % of cardiac arrest

I would like to see the number of Sys <90, sats <90 and HR > 120 rather than the means alone (especially in groups that different by age). The stats should be re run accordingly

Try to shorten the discussion
