# Supplementary material for: Direct transport vs secondary transfer to level I trauma centers in a French exclusive trauma system: Impact on mortality and determinants of triage on road-traffic victims
Source: PLoS One. 2019 Nov 21;14(11):e0223809. doi: 10.1371/journal.pone.0223809 (PMC6872206; doi:10.1371/journal.pone.0223809)
Supplement: Attachment — Submitted filename: Response to Reviewers.docx [file pone.0223809.s006.docx]

**PONE-D-19-19735
Direct transport vs secondary transfer to level I trauma centers in a French exclusive trauma system: impact on mortality and determinants of triage on road-traffic victims
PLOS ONE**

Dear Dr Zsolt J. Balogh,

Academic Editor of PLOS ONE,

Thank you for reviewing and sending back our manuscript " **Direct transport vs secondary transfer to level I trauma centers in a French exclusive trauma system: impact on mortality and determinants of triage on road-traffic victims** ". We did our best to satisfy the comments and we attached a rebuttal letter itemizing each point raised by the reviewers and the academic editor. The changes have been highlighted in blue in the manuscript (“Revised Manuscript with Track Changes”) and a version of our manuscript without tracked change is also available (Manuscript).

We actually have an issue for giving public access to the dataset used for the study. Indeed, The Traumabase is a collaborative project that consists of trauma practitioners, from different centres all over the country, sharing the registry for research and public health issues. The rules of the association are very strict regarding the use of data, and are in accordance with the National Commission for data protection as they contain sensitive information on trauma and outcome and could compromise patients’ confidentiality. We develop these points in the proper paragraph to justify our constraints on sharing the data.

We hope this revised version will be suitable for publication in PLOS ONE. As the corresponding author, I remain at your entire disposal for any further indication.

Sincerely,

Sophie HAMADA, MD, MSc

*sophiehamada@hotmail.com*

**Academic Editor Comments**

1. Please ensure that your manuscript meets PLOS ONE's style requirements, including those for file naming.

The authors report that the manuscript(s) style have been checked to suit PLOS ONE’s requirements. Each file is named as requested and attached to the review.

2. In the *ethics statement in the manuscript* and in the online submission form, please provide additional information about the database used in your retrospective study. Specifically, please ensure that you have discussed whether all data were fully anonymized before you accessed them and/or whether the IRB or ethics committee waived the requirement for informed consent. If patients provided informed written consent to have their data used in research, please include this information.

The Traumabase® obtained approval from the Advisory Committee for Information Processing in Health Research (CCTIRS, 11.305bis) and from the National Commission for data protection (CNIL, 911461), and meets the national institutional review board requirements (Comité de Protection des Personnes Paris VI, Paris France). The information is given to the patients (next of kin) about the existence of the trauma registry, but the institutional review board waived the need for informed consent.

Data are anonymized from the time they are collected in the CRF (computerized record file). Nevertheless, within each center exists a paper file allowing to connect the registry number of the patient to the real patient. This file is kept by the principal investigator of each center, closed in a locked cabinet (as requested by the National Commission for data protection).

So when the investigator access the data of the Traumabase, they are actually anonymized (no name, no initials, and no patient identification number).

In the manuscript Page 7, line 17-20 : “The information is given to the patients (or next of kin, in case of comatose patients) about the existence of the trauma registry, but the institutional review board waived the need for informed consent. Data are anonymized from the time they are collected in the CRF (computerized record file)”.

3. We note that you have indicated that data from this study are available upon request. PLOS only allows data to be available upon request if there are legal or ethical restrictions on sharing data publicly.

a) If there are ethical or legal restrictions on sharing a de-identified data set, please explain them in detail (e.g., data contain potentially identifying or sensitive patient information) and who has imposed them (e.g., an ethics committee). Please also provide contact information for a data access committee, ethics committee, or other institutional body to which data requests may be sent.

The Traumabase is a collaborative project that consists of trauma practitioners, from different centres all over the country, sharing the registry for research and public health issues. The rules of the association are very strict regarding the use of data. Data are only extracted for specific purposes. The leader of a given research project is responsible for the dataset that is extracted from the research unit. No dataset can ever be sent by email or shared in Drop box. Even though datasets are de-identified, the National Commission for data protection (CNIL) has imposed restrictions on data sharing since they contain sensitive information on trauma and outcome and could compromise patients’ confidentiality. Once a project is over, the investigator has the duty to destroy the dataset, and not store the data for personal use or for other projects, not validated by the scientific committee.

In our present study, the Traumabase data have been linked to data owned by the Ministry of the Interior. Restrictions to the sharing of these data are ruled by the body of the Road Safety Office in the Ministry of the Interior and a special convention has been signed (attached, but in French). The right to use these data involved the pledge to secure the data and not to share with people from outside the project.

The scientific committee of the Traumabase can be contacted with the following email address: contact@traumabase.eu (secretary of the scientific committee- **Dr Anatole Harrois**).

b) If there are no restrictions, please upload the minimal anonymized data set necessary to replicate your study findings as either Supporting Information files or to a stable, public repository and provide us with the relevant URLs, DOIs, or accession numbers. Please see http://www.bmj.com/content/340/bmj.c181.long for guidelines on how to de-identify and prepare clinical data for publication. For a list of acceptable repositories, please see http://journals.plos.org/plosone/s/data-availability#loc-recommended-repositories.

Cf previous paragraph

4. One of the noted authors is a group or consortium - The Traumabase Group.

In addition to naming the author group, please list the individual authors and affiliations within this group in the acknowledgments section of your manuscript. Please also indicate clearly a lead author for this group along with a contact email address.

A list of the individual authors and affiliations have been added for each member of the Traumabase Group in the acknowledgment section (Page 30 line 4). The lead author for the group is the first author of the manuscript.

**Questions in the “Manuscript sent back to author” 28/08/2019**

1. Thank you for clarifying that your data is third party data, and cannot be publicly shared.

At this time, please address the following queries:

a) Please confirm that the above proposed statement is both true and correct for your work. If there are any errors or omissions, please note this now.

**We do confirm the following statement:**

"Data are from the Traumabase, a collaborative project that consists of trauma practitioners, from different centres all over the country, sharing the registry for research and public health issues. Even though datasets are de-identified, the National Commission for data protection (CNIL) has imposed restrictions on data sharing since they contain sensitive information on trauma. In the present study, the Traumabase data have been linked to data owned by the Ministry of the Interior. Restrictions to the sharing of these data are ruled by the body of the Road Safety Office in the Ministry of the Interior and a special convention has been signed to secure the data. For data access requests, interested researchers can contact the Traumabase scientific committee with the following email address: [contact@traumabase.eu](mailto:contact@traumabase.eu) (secretary of the scientific committee- Dr Anatole Harrois)."

a) Please clarify whether or not the authors received special access privileges to the data.
The first author received special access privileges to the data, according to a convention signed by the representative of Road Safety delegation within the ministry of interior, the departmental director of the research institute (INSERM) and the president of the Traumabase group (first author), administering the trauma registry.

Here is an extract, translated in English, of the Article 7 of the convention:

“(…) Confidential Information" means all information and/or data in any form and nature whatsoever - including, in particular, written or printed documents, samples, models and/or knowledge, whether patentable or not, patented or not, provided by a Party to one or more other Parties under this Agreement, for which the Party providing such information has unequivocally indicated its confidential nature.

Each Party undertakes to keep confidential all Information communicated by another Party, and declared as such by that Party, in execution or in connection with this Agreement. (…)

In this respect, the parties agree that communicating or making available the information that one of them would qualify as Confidential information due to the personal nature of the data is not authorized (…)”.

The Road Safety delegation, through its representative, clearly indicated the confidential nature of its data. Consequently, INSERM and TRAUMABASE® are bound by the obligation of discretion with regard to all available information brought to their attention by the Road Safety delegation during the execution of this agreement.

Regarding the Traumabase registry, the rule of data accessibility requires a pathway through two different committees: the “Scientific Committee” and the “Data Protection and Quality Governance” to assess the quality of the project, to identify which data have to be extracted and to protect the dispensing of the “material” data (encoding, crypting, delivering). This latter point was deemed to be mandatory by the National Commission for data protection (CNIL) to ensure data confidentiality.

Thus, combining those two sources of data for the present study involved a large preliminary administrative work so that the first author had the privilege to gain access to both dataset. Hence, the authors are bound by the terms of the signed convention clearly stipulating that data cannot be communicated.

b) Please confirm that interested researchers will be able to replicate the results of your study by following the protocol outlined in the Methods section.

We assure and confirm that interested researchers would be able to replicate the results of the present study, by following the protocol described in the method section.

**Reviewers' comments (attachment file):**

Trauma data base retrospective analysis of direct versus secondary road trauma admissions.

Primary outcome: mortality 30 days

English: good

Aim: interesting

Limits acknowledged.

My main concern is the small number of patients who died (primary outcome) in secondary admissions group (9!). A statistical consultant opinion is warranted to ensure these results are sound and assessable.

The results of the present study are the process of the analysis of a 3 years long cohort from 6 trauma centers of the Ile de France region. The number of death within the group “Secondary transfer” is low, but is the product of an exhaustive observation of the real life. This study has been produced in a research center in public health (“CESP”: Reseach Center in Epidemiology and Population Health from the National Institute in Health and medical research, INSERM) with epidemiologists and statisticians. The results have been validated by graduated statisticians. In the multivariate logistic regression model assessing the association between mortality (i.e. the dependent variable; 278 events) and secondary transfer (4031 vs 381 patients), there are 18 independent variables which is lower than the number of independent variables allowed based on the 10 events by variable rule, collinearity between independent variables has been checked and linearity between continuous independent variables and log odds has been checked. Also, model calibration was assessed using the Hosmer and Lemeshow statistic, model discrimination was assessed using the area under the ROC curve and optimism was evaluated using a bootstrap methodology as recommended in the Transparent Reporting of a multivariable prediction model for Individual Prognosis Or Diagnosis (TRIPOD) statement (Moons KGM, Altman DG, Reitsma JB, et al (2015) Transparent Reporting of a multivariable prediction model for Individual Prognosis Or Diagnosis (TRIPOD): Explanation and Elaboration. Annals of Internal Medicine 162:W1. doi: 10.7326/M14-0698). So even with 9 (7%) death in one group, validity condition to implement the model were checked and are respected.

Suggestions:

- Abstract

1. Define groups in methods and do not explain again in results

The groups are defined in page 8, line 23-24 “The modality of admission was defined as *direct transport* if the patient was directly transported from the scene to a level 1 TC, and as *secondary transfer* if the patient was transferred to a level 1 TC within the first 48 hours after the RTA but after a previous admission in a lower level facility.”

Repeated precisions about the groups were withdrawn in the results paragraph (page 11 - line 15; page 15 – line 2).

2. Mention confounders

We explain in the “*statistical analysis*” section that the confounders were identified with the univariate analyses for transport modality and for mortality, and selected if they were statistically associated with the chosen outcome.

Page 10, lines 17_20

“All confounders, i.e. the variables associated with the outcomes (p-value <0.05) in univariate analyses, were selected for multivariate analyses except in case of collinear variables (Spearman correlation coefficients >0.8), where the most clinically relevant variable was selected (27).”

3. Define undertriage in methods and report in results

We withdrew the re-explanation of the systemic undertriage in the result section (Page 13, line 6-7) and focused on the definitions of undertriage in the method section.

Page 9, line 20 : “Undertriage, defined as severe patients not ending in a TC, was computed according to three approaches: (…)”

**- Body**

3.MGAP has been introduced twice

This has been corrected in the manuscript (Page 9, line 8)

**- In results:**

4. Add number and % of deaths in both groups. It is relevant as the number is secondary transfer group are really small.

We added the numbers and % in the sentence Page 12, line 20

“While crude mortality was significantly lower when patients were secondarily transferred (2% (n=9) vs 7% (n=269), p < 0.001)”.

5. Again stick to primary and secondary admission group. It is too often re introduced

Repeated precisions about the groups were withdrawn in the results paragraph (page 11 - line 21; page 15 – line 8).

6. There is a typo in % of cardiac arrest

The typing error has been corrected in the Table 2, line “Traumatic cardiac arrest”.

7. I would like to see the number of Sys <90, sats <90 and HR > 120 rather than the means alone (especially in groups that different by age). The stats should be re-run accordingly

a) Table including the requested dichotomization of physiologic quantitative variables

SAP systolic arterial pressure, HR : heart rate, SpO2 : peripheral oxygen saturation

b) As stated in the Table 2, initial data for HR, SAP and SpO2 were missing, not at random in patients with secondary transfer (missing SAP 22.8%, HR 22.6% and SpO2 25.7%). In direct transport, those same variables were missing only for: SAP 1.2%, HR 1.4%, Spo2 3.1%.

So in our study, we made the argued choice of withdrawing prehospital SAP from the model because of the missing values in the secondary transfer group.

c) Argumentation:

Indeed, in a sensitivity analysis with only “complete cases” for these prehospital physiologic variables (3868 patients in the direct group and 262 in the secondary transfer), SAP remained in the final model but not HR and SpO2. As it can be seen in the tables below (Table 4 and table 4bis), the values of the OR of all the identified predictors were marginally modified in this sensitivity analyses. Data missing not at random are known to introduce bias, so we chose to withdraw these prehospital variables and we showed that it had no meaningful impact on our results with these sensitivity analyses.

Table 4 and 4 bis (Supplemental material).

d) And to finish, to enlighten our choice about the 3 quantitative variables listed by the reviewer, they have been processed to be the most accurate in the complete case model (sensitivity analysis). Indeed, previous statistical studies have indicated that dichotomizing a continuous confounding variable in multivariate regression analyses can lead to biased estimation of the effect of exposure on outcomes. So, prior to entry a qualitative variable in the multivariate analysis, we tested whether or not the continuous confounding variable was linearly related to log-odds of the binary outcome. Dichotomization (as requested by the reviewer) assumes that the relationship between the predictor and the outcome is flat within intervals, i.e., a discontinuity in outcome as interval boundaries are crossed [Altman DG, Royston P (2006) The cost of dichotomising continuous variables. *British Medical Journal* 332:1080]. We tested this hypothesis using restricted cubic spline for our data. For SAP (left) and HR (right), the relationship was rather linear than discontinuous.

When it came to saturation, not only this variable was not significantly associated with transport on univariate analysis, but it also showed a neighboring discontinuous relation, but rather on a cut-off around 80%.

So, finally we added 3 lines in the Table 2 to integrate the reviewer demand, and added the precision about the sensitivity analysis on the model. We intended to remain both clear and not too cumbersome for the readers.

Page 10, line 20-23: “Initial physiologic variables (arterial pressure, heart rate and oxygen saturation) were withdrawn from the model because they were missing not at random in the secondary transfer group for more than 22% (vs <2% in the direct transport group).”

Page 11, line 9 “A sensitivity analysis was performed with a model on “complete cases” gathering all prehospital physiologic variables.”

Page 12, line 15-16: “In the sensitivity analysis, the values of the OR of all the identified predictors were marginally modified by prehospital physiologic variables.”

8. Try to shorten the discussion

Before shortening the discussion was 1442 words long.

After improvement, we ended with 1320 words in the discussion. The changes are visible in the manuscript with track changes.

**Reviewers' comments to the authors (computerized file):**

1. Is the manuscript technically sound, and do the data support the conclusions?
Reviewer #1: Partly
Reviewer #2: Yes

2. Has the statistical analysis been performed appropriately and rigorously? 
Reviewer #1: I Don't Know
Reviewer #2: Yes

We reassured the reviewer in the first paragraph of the “Reviewers comment (attachment file)”.

3. Have the authors made all data underlying the findings in their manuscript fully available?
Reviewer #1: No
Reviewer #2: No

These elements are developed in the “Academic editor comments”, paragraph 3.a).

4. Is the manuscript presented in an intelligible fashion and written in standard English?
Reviewer #1: Yes
Reviewer #2: Yes

5. Review Comments to the Author

5.1. Reviewer #1: needs a good statistician.
Not convinced by numbers.
otherwise a nice paper (maybe prehospital J might be more interested

This study has been produced in a research center in public health (“CESP”: Reseach Center in Epidemiology and Population Health from the National Institute in Health and medical research, INSERM) with epidemiologists and statisticians. The results have been validated by graduated statisticians (cf paragraph “Reviewers comments (attachment file)”).

5.2. Reviewer #2: Interesting study from a well developed trauma system in Paris.

My only major comment is that I don't get a sense of the total number of non trauma centres in that region, so the comment regarding "exclusive" trauma system is somewhat intriguing. Inclusive or exclusivity really depends on the proportion of major tertiary hospitals that are designated trauma centres (Level 1 or 2), so it may be argued that having 6 TCs in central Paris is really indicative of an inclusive system, depending on the number of similar sized tertiary hospitals within Paris.

In the “exclusive trauma system” type, patients are referred to a low number of very specialized and designated hospitals, the so called “Trauma Center”. Severe trauma management is conceived only in those facilities, trauma center being most of the time university hospitals. An exclusive system functions as a funnel, not a network, and it does not use the resources of other health-care facilities within the region. This is the case in “Île de France” region, as presented on the Figure 1. There are eighty-seven public hospitals gathering medical, surgical and obstetrical care (tertiary care centers) within the region of which 6 are labelled “trauma centers”.

Page 6, line 18-19: Within the legend of Figure 1. “The region is organized as an exclusive trauma system, conceiving severe trauma care only in the 6 designated TC among all existing tertiary care facilities (n=87).”

The only other major issue is survivor bias for patients undergoing secondary transfer and the need to report matched mortality for patients not transferred to the TC. The authors have identified this I think, but some discussion on the need for an integrated database across all facilities, non trauma and trauma centres, is warranted given the volume of trauma being reported.

We thank the reviewer for this very important comment. We are actually preparing a very large study to actively monitor all those patients arriving (and dying) in non-specialized centers; and comparing inclusive and exclusive trauma systems in France.

Page 23, line 5-7: “A large observational study in France is about to begin and will allow to have an integrated database across all facilities of the participating regions to answer this point.”

Lastly, I think a recent paper by Cornwall et al Injury 2019 on trauma transfers within a network in Australia outlining similar results would be worthwhile to place these results in context

We thank the reviewer for this proposition. We actually added this reference in the discussion of the manuscript.

Page 23, line 7-9: “An Australian study even showed that, in a well-resourced and coordinated trauma network, mortality was lower in non-TC when adjusted for age and injury severity (46).”
